# Reduced Insulin Signaling Targeted to Serotonergic Neurons but Not Other Neuronal Subtypes Extends Lifespan in *Drosophila melanogaster*

**DOI:** 10.3389/fnagi.2022.893444

**Published:** 2022-07-05

**Authors:** Nikolett Dravecz, Tommy Shaw, Isabella Davies, Casey Brown, Lewis Ormerod, Gin Vu, Tyler Walker, Taran Taank, Alan D. Shirras, Susan J. Broughton

**Affiliations:** Division of Biomedical and Life Sciences, Faculty of Health and Medicine, Lancaster University, Lancaster, United Kingdom

**Keywords:** ageing, behavioral senescence, insulin/IGF-like signaling, serotonergic neurons, *Drosophila*

## Abstract

Reduced Insulin/IGF-like signaling (IIS) plays an evolutionarily conserved role in improving longevity and some measures of health-span in model organisms. Recent studies, however, have found a disconnection between lifespan extension and behavioral health-span. We have previously shown that reduction of IIS in *Drosophila* neurons extends female lifespan but does not improve negative geotaxis senescence and has a detrimental effect on exploratory walking senescence in both sexes. We hypothesize that individual neuronal subtypes respond differently to IIS changes, thus the behavioral outcomes of pan-neuronal IIS reduction are the balance of positive, negative and neutral functional effects. In order to further understand how reduced IIS in neurons independently modulates lifespan and locomotor behavioral senescence we expressed a dominant negative Insulin receptor transgene selectively in individual neuronal subtypes and measured the effects on lifespan and two measures of locomotor senescence, negative geotaxis and exploratory walking. IIS reduction in cholinergic, GABAergic, dopaminergic, glutamatergic, and octopaminergic neurons was found to have either no affect or a detrimental effect on lifespan and locomotor senescence. However, reduction of IIS selectively in serotonergic neurons resulted in extension of lifespan in females with no effect on locomotor senescence. These data indicate that individual neuronal subtypes respond differently to IIS changes in the modulation of lifespan and locomotor senescence, and identify a specific role for the insulin receptor in serotonergic neurons in the modulation of lifespan.

## Introduction

Alteration of IIS can have pleiotropic effects on growth, development, metabolic homoeostasis, reproduction and aging (Kimura et al., 1997; Brogiolo et al., 2001; Clancy et al., 2001; Saltiel and Kahn, 2001; Ikeya et al., 2002; Rulifson et al., 2002; Martin and Grotewiel, 2006; Rhodenizer et al., 2008; Selman et al., 2008). There is thus much focus on the pathway as an evolutionarily conserved modulator of lifespan and age-related health in nematode worms, fruit flies and mice. However, not all of these IIS-related lifespan extending mutations ameliorate the age-related decline of functional health and there is still much we do not understand about the relationships between IIS, lifespan and health-span. In particular, we still know little about the role of IIS in aging and function of the central nervous system (CNS). Given the diverse roles that IIS plays in the CNS it is possible that reductions in it will have positive or negative effects on neuronal survival and function (Broughton and Partridge, 2009). For instance, IIS is involved in learning and memory (Chambers et al., 2015), sleep (Cong et al., 2015), and PI3K activity is required for synaptogenesis and synapse maintenance in the adult (Martin-Pena et al., 2006). It is not surprising, therefore, that studies in model organisms have found a disconnection between functional senescence and lifespan extension due to genetic or environmental interventions (Cook-Wiens and Grotewiel, 2002; Burger and Promislow, 2006; Bhandari et al., 2007; Burger et al., 2008).

In *Drosophila* we have previously shown that the insulin receptor in neurons independently modulates lifespan and different types of locomotor senescence (Ismail et al., 2015). We investigated the role of neural IIS in locomotor senescence by measuring the age-related performance of two different locomotor behaviors—negative geotaxis and exploratory walking—in *Drosophila melanogaster* with ubiquitous or neural-specific IIS reductions. Our data showed that reduced IIS can be detrimental to CNS function even when the reduction is sufficient to result in extension of lifespan (Ismail et al., 2015). This disconnection between lifespan and behavioral health-span raises questions about the role of IIS in the brain on organismal aging, brain aging, and brain function. In this study we aimed to further our understanding of this uncoupling of lifespan and behavioral senescence in response to alterations in neural IIS in *Drosophila*, by investigating the role of IIS in different neuronal subtypes in the modulation of lifespan and locomotor senescence. Given the neural control of behavior and the variable rates and ages of onset of behavioral declines in function during aging (Grotewiel et al., 2005) it is likely that manipulating IIS in the CNS will have diverse effects on the aging and/or function of the neural circuitries underlying different behaviors. Moreover, it is possible that the effects of pan-neural IIS knockdown seen in Ismail et al. (2015) were the result of the balance of positive, negative or neutral effects on different neuronal subtypes across the CNS. To determine which neuronal subtypes play a role in modulating lifespan and locomotor behavioral senescence in response to altered IIS, the UAS-InR^DN^ transgene (Ikeya et al., 2009; Ismail et al., 2015) was targeted to specific neuronal subtypes and effects on lifespan, negative geotaxis and exploratory walking were measured. IIS reductions in cholinergic, GABAergic, dopaminergic, glutamatergic, serotonergic, and octopaminergic neurons were found to have variable effects on lifespan and locomotor senescence and there was little correlation between an effect on lifespan and an effect on locomotor senescence. Notably, reduction of IIS specifically in serotonergic neurons resulted in extension of lifespan in females with no effect on locomotor senescence. Together, the data indicate that individual neuronal subtypes respond differently to changes in IIS, suggesting that the effect of pan-neural IIS reduction on lifespan and locomotor senescence was due to the sum of effects on different neuronal subtypes. The data further support previous findings of a disconnection between lifespan and behavioral health-span and identify a specific role for IIS in serotonergic neurons for the modulation of lifespan.

## Materials and Methods

### Fly Stocks and Maintenance

All fly stocks were initially backcrossed at least 5 times into the white^Dahomey^ (w^Dah^) outbred background, as previously described (Broughton et al., 2005), and re-backcrossed regularly including just prior to each analysis. UAS-InR^DN^ is described in Ikeya et al. (2009); briefly—UAS-dInRA1409K (chr. II) (denoted here as UAS-InR^DN^) was obtained from the Bloomington Drosophila Stock Centre (ref. FBal015635). The UAS-InR^DN^ transgene causes an amino acid substitution in the kinase domain (R1409A) of the Drosophila insulin receptor (dInR), resulting in its dominant negative activity. Targeting of the UAS-InR^DN^ transgene was achieved using the following neuronal subtype-specific GAL4 lines: Th-GAL4 (dopaminergic neurons), Vglut-GAL4 (glutamatergic neurons), Chat-GAL4 (cholinergic neurons), Gad1-GAL4 (GABAergic neurons) trhGAL4 (serotonergic), and TdcGAL4 (octopaminergic). The following GAL4 lines were obtained from the Bloomington Drosophila Stock Centre: elavGAL4^*C*155^ (#458), TrhGAL4 (#38389), TdcGAL4 (#9313), ThGAL4 (#8848), VglutGAL4 (#26160), ChATGAL4 (#6798), and Gad1GAL4 (#51630). Stocks were maintained and experiments conducted at 25°C on a 12 h:12 h light:dark cycle at constant humidity using standard sugar/yeast medium [100 g/L brewer’s yeast (MP Biomedicals), 50 g/L sucrose, 10 g/L agar] (Bass et al., 2007). Flies for all experiments were reared at standard larval density, as previously described (Broughton et al., 2005). Eclosing adults were collected over a 12 h period and mated for 48 h before sorting into single sexes.

### Lifespan

Procedures for lifespan studies are as described in Clancy et al. (2001). Lifespan was measured in once mated female or male flies kept at 10/vial on standard food medium and transferred to new food three times a week. Deaths were scored once per day 5–6 times per week.

### Locomotor Behavior

Each behavioral experiment (negative geotaxis and exploratory walking) was carried out at 25°C in parallel with survival analysis of separate cohorts of flies generated and maintained under the same conditions.

#### Negative Geotaxis

Negative geotaxis of males and females was measured as described in Rival et al. (2004) and Kerr et al. (2011) at weekly intervals throughout the lifespan. Briefly, 15 adult flies were placed in a vertical column (25 cm long, 1.5 cm diameter) and allowed to recover for 30 min. Flies were tapped to the bottom of the column, and flies reaching the top of the column or remaining at the bottom after a 45 s period were counted. Three trials were performed at 1 min intervals for each experiment. The mean number of flies at the top (*n*top), the mean number of flies at the bottom (*n*bottom) and the total number of flies assessed (*n*tot) were recorded. Performance index was calculated as 1/2(*n*tot + *n*top −*n*bottom)/*n*tot, as described in Rival et al. (2004).

#### Exploratory Walking

Exploratory walking behavior of individual male or female flies was measured in 4 cm diameter/1 cm height circular Perspex arenas as described in Ismail et al. (2015). Chambers contained 4 arenas such that 4 flies could be videoed simultaneously. Individual flies were aspirated into each arena, allowed to rest for 1 min and then were videoed for 15 min. Videos were analyzed using Ethovision XT video tracking software (Noldus), as described in Martin (2004) and Ismail et al. (2015). The walking behavior of flies was measured in this way every 10 days throughout the lifespan. As shown in Ismail et al. (2015), exploratory walking is a complex locomotor behavior that shows a stereotypical pattern of behaviors with robust aging-related changes. Walking parameters including distance walked, speed and duration of walking and number of rotations (changes in walking direction) all reduce with age, whilst other parameters such as time spent in the central zone and the latency to the first change of walking direction increase with age. Whilst walking velocity and distance can indicate general neuromuscular health and function, other parameters are indicative of decision making (walking duration, rotation frequency, latency to first rotation, duration in central zone) and hence can be used to indicate a decline in the neural control of behavior with age.

### Statistical Analyses

Statistical analyses were performed using JMP (version 8) software (SAS Institute). Lifespan data were subjected to survival analysis (Log Rank tests) and presented as survival curves. Other data (QPCR and locomotor behavior) were tested for normality using the Shapiro-Wilk W test on studentized residuals (Sokal and Rohlf, 2011) and appropriately transformed where necessary. Two-way (genotype and age) or one-way (genotype) analyses of variance (ANOVA) were performed and planned comparisons of means were made using Tukey-Kramer HSD, *p* < 0.05. Data are presented as means of raw data ± SEM and * denotes significant difference from controls.

## Results

### Reduced Insulin/IGF-Like Signaling in Serotonergic Neurons Is Sufficient to Extend Female Lifespan

**FIGURE 1:**
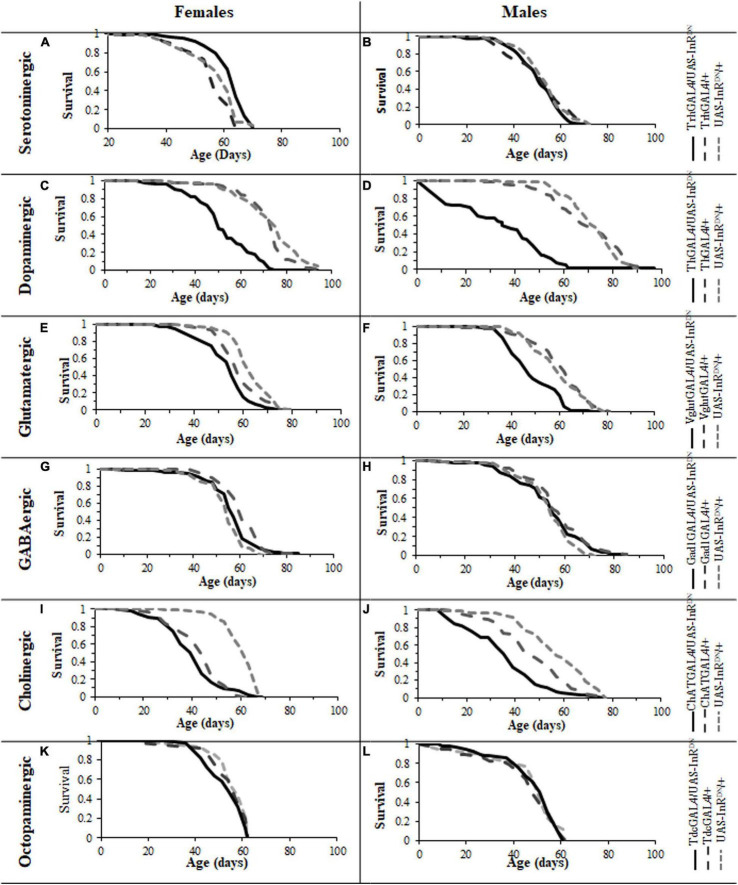
Survival of male and female flies with neuron specific (GAL4/UAS-InR^DN^) reductions in IIS. **(A)** Survival of TrhGAL/UAS-InR^DN^ once mated female flies compared to TrhGAL/+ and UAS-InR^DN^/ + controls. Median lifespans and sample sizes were: TrhGAL/UAS-InR^DN^ = 64 days, *N* = 125; TrhGAL/ + = 57 days, *N* = 112; and UAS-InR^DN^/+ = 61 days, *N* = 113. TrhGAL/UAS-InR^DN^ females showed an increased survival compared to both controls by log rank tests (*P* < 0.0001). **(B)** Survival of TrhGAL/UAS-InR^DN^ male flies compared to TrhGAL/ + and UAS-InR^DN^/ + controls. Median lifespans and sample sizes were: TrhGAL/UAS-InR^DN^ = 51 days, *N* = 91; TrhGAL/ + = 54 days, *N* = 87; d UAS-InR^DN^/ + = 54 days, *N* = 87. **(C)** Survival of ThGAL/UAS-InR^DN^ once mated female flies compared to ThGAL/ + and UAS-InR^DN^/ + controls. Median lifespans and sample sizes were: ThGAL/UAS-InR^DN^ = 49 days, *N* = 150; ThGAL/ + = 73.5 days, *N* = 148; and UAS-InR^DN^/ + = 73.5 days, *N* = 144. ThGAL/UAS-InR^DN^ females showed a decreased survival compared to both controls by log rank tests (*P* < 0.001). **(D)** Survival of ThGAL/UAS-InR^DN^ male flies compared to ThGAL/ + and UAS-InR^DN^/ + controls. Median lifespans and sample sizes were: ThGAL/UAS-InR^DN^ = 34 days, *N* = 149; ThGAL/ + = 73.1 days, *N* = 153; and UAS-InR^DN^/ + = 74.5 days, *N* = 153. ThGAL/UAS-InR^DN^ males showed a decreased survival compared to both controls by log rank tests (P = ??). **(E)** Survival of VglutGAL/UAS-InR^DN^ once mated female flies compared to VglutGAL/ + and UAS-InR^DN^/ + controls. Median lifespans and sample sizes were: VglutGAL/UAS-InR^DN^ = 54 days, *N* = 100; VglutGAL/ + = 56.5 days, *N* = 100; and UAS-InR^DN^/ + = 61 days, *N* = 100. VglutGAL/UAS-InR^DN^ females showed a decreased survival compared to both controls by log rank tests (*P* = 0.0008 to VglutGAL4/ + and *P* < 0.0001 to UAS-InR^DN^/ +). **(F)** Survival of VglutGAL/UAS-InR^DN^ male flies compared to VglutGAL/ + and UAS-InR^DN^/ + controls. Median lifespans and sample sizes were: VglutGAL/UAS-InR^DN^ = 45 days, *N* = 100; VglutGAL/ + = 61 days, *N* = 100; and UAS-InR^DN^/ + = 59 days, *N* = 100. VglutGAL/UAS-InR^DN^ males showed a decreased survival compared to both controls by log rank tests (*P* < 0.0001). **(G)** Survival of Gad1GAL/UAS-InR^DN^ once mated female flies compared to Gad1GAL/ + and UAS-InR^DN^/ + controls. Median lifespans and sample sizes were: Gad1GAL/UAS-InR^DN^ = 56 days, *N* = 148; Gad1GAL/ + = 59 days, *N* = 149; and UAS-InR^DN^/ + = 54 days, *N* = 154. **(H)** Survival of Gad1GAL/UAS-InR^DN^ male flies compared to Gad1GAL/ + and UAS-InR^DN^/ + controls. Median lifespans and sample sizes were: Gad1GAL/UAS-InR^DN^ = 54 days, *N* = 148; Gad1GAL/ + = 54 days, *N* = 148; and UAS-InR^DN^/ + = 54 days, *N* = 161. **(I)** Survival of ChATGAL/UAS-InR^DN^ once mated female flies compared to ChATGAL/ + and UAS-InR^DN^/ + controls. Median lifespans and sample sizes were: ChATGAL/UAS-InR^DN^ = 41.5 days, *N* = 100; ChATGAL/ + = 41.5 days, *N* = 102; and UAS-InR^DN^/ + = 61 days, *N* = 99. ChATGAL/UAS-InR^DN^ and ChATGAL/ + females showed a decreased survival compared to the UAS-InR^DN^/ + control females by log rank tests (*P* < 0.0001). **(J)** Survival of ChATGAL/UAS-InR^DN^ male flies compared to ChATGAL/ + and UAS-InR^DN^/ + controls. Median lifespans and sample sizes were: ChATGAL/UAS-InR^DN^ = 33.5 days, *N* = 100; ChATGAL/ + = 46 days, *N* = 100; and UAS-InR^DN^/ + = 55 days, *N* = 100. ChATGAL/UAS-InR^DN^ males showed a decreased survival compared to both controls by log rank tests (*P* < 0.0001). **(K)** Survival of TdcGAL/UAS-InR^DN^ once mated female flies compared to TdcGAL/ + and UAS-InR^DN^/ + controls. Median lifespans and sample sizes were: TdcGAL/UAS-InR^DN^ = 60 days, *N* = 153; TdcGAL/ + = 60 days, *N* = 153; and UAS-InR^DN^/ + = 60 days, *N* = 125. **(L)** Survival of TdcGAL/UAS-InR^DN^ male flies compared to TdcGAL/ + and UAS-InR^DN^/ + controls. Median lifespans and sample sizes were: TdcGAL/UAS-InR^DN^ = 54 days, *N* = 71; TdcGAL/ + = 51 days, *N* = 84; and UAS-InR^DN^/ + = 51 days, *N* = 40.

Expression of the UAS-InR^DN^ transgene restricted to specific neuronal subtypes resulted in varying effects on lifespan (Figure 1). As shown in Figures 1A,B, a constitutive reduction of IIS specifically in serotonergic neurons was sufficient to extend lifespan in females, with no effect on male lifespan. Interestingly, a similar sex-specific effect was seen with pan-neural IIS reduction (Ismail et al., 2015). The lifespan extending effect in females was confirmed in two repeat lifespan experiments (Supplementary Figure 1).

### Reduced Insulin/IGF-Like Signaling in Other Neuronal Subtypes Has a Negative or No Impact on Lifespan

In contrast, expression of UAS-InR^DN^ in other neuronal subtypes either had no effect on lifespan or resulted in a reduction in lifespan (Figure 1). In this way, reduced IIS in dopaminergic neurons (ThGAL4/UAS-InR^DN^) significantly reduced the lifespan of both male and female flies (*p* < 0.001) (Figures 1C,D) by approximately 50 and 30%, respectively. Similarly, glutamatergic neuron specific expression of UAS-InR^DN^ (VglutGAL4/UAS-InR^DN^) resulted in a significant reduction in lifespan of approximately 10% in females (*p* = 0.0008 to VglutGAL4/ + and *p* < 0.0001 to InR^DN^/ +) and 25% in males (*p* < 0.0001) (Figures 1E,F). However, the VglutGAL4 driver itself had a negative effect on female lifespan as the VglutGAL4/ + control group had a significantly shorter lifespan compared to the UAS-InR^DN^/ + control group. Reduced IIS in cholinergic neurons (ChATGAL4/UAS-InR^DN^) had no significant effect on female lifespan (Figure 1I) and resulted in a shortening of male lifespan (*P* < 0.0001) (Figure 1J). However, similarly to the VglutGAL4 driver, the ChATGAL4 driver induced a significantly shorter lifespan than the UAS-InR^DN^/ + control. Expression of UAS-InR^DN^ in GABAergic neurons (GadGAL4/UAS-InR^DN^, Figures 1G,H) and octopaminergic neurons (TdcGAL4/UAS-InR^DN^, Figures 1K,L) had no effect on male or female lifespan.

Together, these data suggest that the lifespan extension of female *Drosophila* resulting from pan-neural IIS reduction is due to the sum of varying effects of reduced IIS in different neuronal subtypes. Furthermore, the data show that reduced IIS specifically in serotonergic neurons is sufficient to extend female lifespan, identifying a specific role for these neurons in the extension of lifespan in response to reduced IIS.

### Reduced Insulin/IGF-Like Signaling in Serotonergic Neurons Does Not Improve Locomotor Behavior Senescence

**FIGURE 2:**
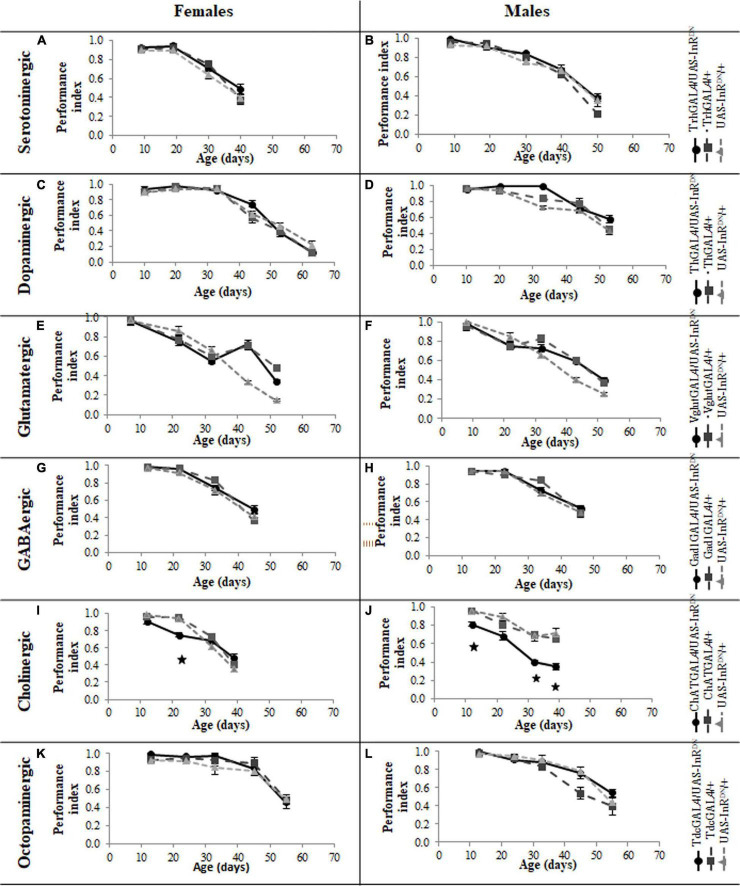
Effect of constitutive IIS reduction in specific neuronal subtypes on negative geotaxis senescence. **(A)** Negative geotaxis performance index over the lifespan of TrhGAL4/UAS-InR^DN^ once mated female flies compared to TrhGAL4/ + and UAS-InR^DN^/ + controls, *N* = 3 (groups of 10 flies) for each genotype. **(B)** Negative geotaxis performance index over the lifespan of TrhGAL4/UAS-InR^DN^ male flies compared to TrhGAL4/ + and UAS-InR^DN^/ + controls, *N* = 3 (groups of 10 flies) for each genotype. **(C)** Negative geotaxis performance index over the lifespan of ThGAL4/UAS-InR^DN^ once mated female flies compared to ThGAL4/ + and UAS-InR^DN^/ + controls, *N* = 3 (groups of 10 flies) for each genotype. **(D)** Negative geotaxis performance index over the lifespan of ThGAL4/UAS-InR^DN^ male flies compared to ThGAL4/ + and UAS-InR^DN^/ + controls, *N* = 3 (groups of 10 flies) for each genotype. **(E)** Negative geotaxis performance index over the lifespan of VglutGAL4/UAS-InR^DN^ once mated female flies compared to VglutGAL4/ + and UAS-InR^DN^/ + controls, *N* = 3 (groups of 10 flies) for each genotype. **(F)** Negative geotaxis performance index over the lifespan of VglutGAL4/UAS-InR^DN^ male flies compared to VglutGAL4/ + and UAS-InR^DN^/ + controls, *N* = 3 (groups of 10 flies) for each genotype. **(G)** Negative geotaxis performance index over the lifespan of GAD1GAL4/UAS-InR^DN^ once mated female flies compared to GAD1GAL4/ + and UAS-InR^DN^/ + controls, *N* = 3 (groups of 10 flies) for each genotype. **(H)** Negative geotaxis performance index over the lifespan of GAD1GAL4/UAS-InR^DN^ male flies compared to GAD1GAL4/ + and UAS-InR^DN^/ + controls, *N* = 3 (groups of 10 flies) for each genotype. **(I)** Negative geotaxis performance index over the lifespan of ChATGAL4/UAS-InR^DN^ once mated female flies compared to ChATGAL4/ + and UAS-InR^DN^/ + controls, *N* = 3 (groups of 10 flies) for each genotype. At the age of 22 days ChATGAL4/UAS-InR^DN^ showed decreased negative geotaxis response compared to the controls (*P* = 0.0001 to ChATGAL4/ + and *P* = 0.0003 to UAS-InR^DN^/ +) **(J)** Negative geotaxis performance index over the lifespan of ChATGAL4/UAS-InR^DN^ male flies compared to ChATGAL4/ + and UAS-InR^DN^/ + controls, *N* = 3 (groups of 10 flies) for each genotype. At the age of 12, 32, and 39 days ChATGAL4/UAS-InR^DN^ showed decreased negative geotaxis response compared to the controls (12 days: *P* = 0.0169 to ChATGAL4/ + and *P* = 0.0160 to UAS-InR^DN^/ +; 32 days: *P* = 0.0133 to ChATGAL4/ + and *P* = 0.0164 to UAS-InR^DN^/ +; 39 days: *P* = 0.0281 to ChATGAL4/ + and *P* = 0.0134 to UAS-InR^DN^/ +) **(K)** Negative geotaxis performance index over the lifespan of TdcGAL4/UAS-InR^DN^ once mated female flies compared to TdcGAL4/ + and UAS-InR^DN^/ + controls, *N* = 3 (groups of 10 flies) for each genotype. **(L)** Negative geotaxis performance index over the lifespan of TdcGAL4/UAS-InR^DN^ male flies compared to TdcGAL4/ + and UAS-InR^DN^/ + controls, *N* = 3 (groups of 10 flies) for each genotype.

**FIGURE 3:**
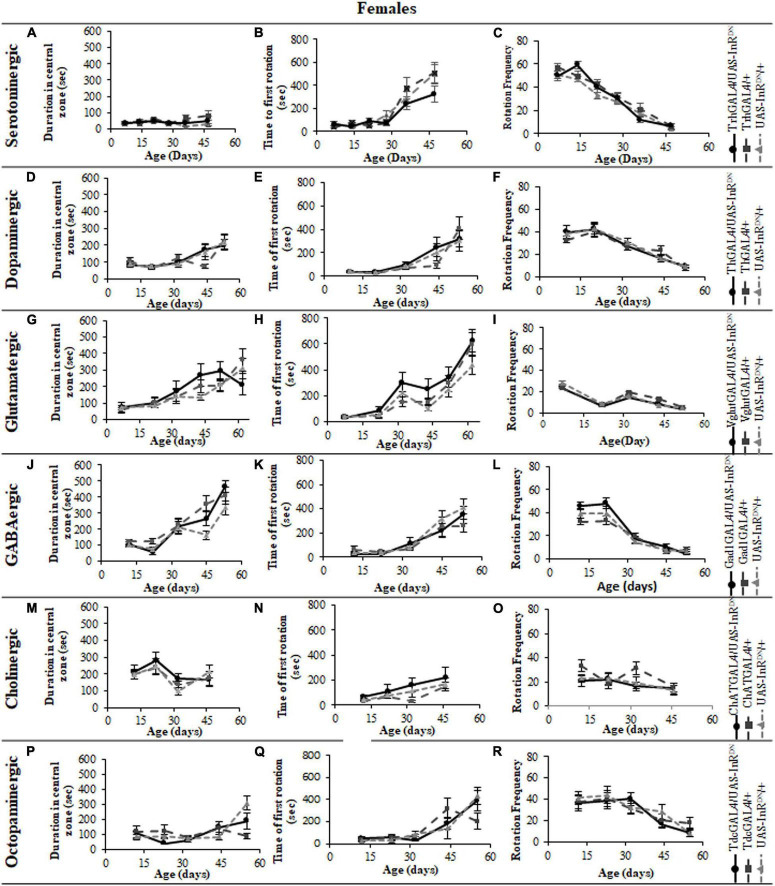
The effect of neuronal subtype specific IIS reduction on locomotion during exploratory walking senescence in female flies. Exploratory walking senescence parameters for a cohort of once-mated female flies of the indicated genotypes run in parallel with the survival experiments shown in Figure 1. Data are shown as mean value for each parameter ± SEM, *N* = 16 for the indicated genotype. Data were analyzed by two-way ANOVA (age and genotype) and age was found to be a significant effect (*p* < 0.05) for all genotypes. **(A)** Mean total distance walked vs. age of TrhGAL4/UAS-InR^DN^ flies compared to TrhGAL4/ + and UAS-InR^DN^/ + controls. **(B)** Mean duration of walking vs. age of TrhGAL4/UAS-InR^DN^ flies compared to TrhGAL4/ + and UAS-InR^DN^/ + controls. **(C)** Mean speed of walking vs. age of TrhGAL4/UAS-InR^DN^ flies compared to TrhGAL4/ + and UAS-InR^DN^/ + controls. **(D)** Mean total distance walked vs. age of ThGAL4/UAS-InR^DN^ flies compared to ThGAL4/ + and UAS-InR^DN^/ + controls. **(E)** Mean duration of walking vs. age of ThGAL4/UAS-InR^DN^ flies compared to ThGAL4/ + and UAS-InR^DN^/ + controls. **(F)** Mean speed of walking vs. age of ThGAL4/UAS-InR^DN^ flies compared to ThGAL4/ + and UAS-InR^DN^/ + controls. **(G)** Mean total distance walked vs. age of VglutGAL4/UAS-InR^DN^ flies compared to VglutGAL4/ + and UAS-InR^DN^/ + controls. **(H)** Mean duration of walking vs. age of VglutGAL4/UAS-InR^DN^ flies compared to VglutGAL4/ + and UAS-InR^DN^/ + controls. **(I)** Mean speed of walking vs. age of VglutGAL4/UAS-InR^DN^ flies compared to VglutGAL4/ + and UAS-InR^DN^/ + controls. **(J)** Mean total distance walked vs. age of Gad1GAL4/UAS-InR^DN^ flies compared to Gad1GAL4/ + and UAS-InR^DN^/ + controls. **(K)** Mean duration of walking vs. age of Gad1GAL4/UAS-InR^DN^ flies compared to Gad1GAL4/ + and UAS-InR^DN^/ + controls. **(L)** Mean speed of walking vs. age of Gad1GAL4/UAS-InR^DN^ flies compared to Gad1GAL4/ + and UAS-InR^DN^/ + controls. **(M)** Mean total distance walked vs. age of ChATGAL4/UAS-InR^DN^ flies compared to ChATGAL4/ + and UAS-InR^DN^/ + controls. **(N)** Mean duration of walking vs. age of ChATGAL4/UAS-InR^DN^ flies compared to ChATGAL4/ + and UAS-InR^DN^/ + controls. **(O)** Mean speed of walking vs. age of ChATGAL4/UAS-InR^DN^ flies compared to ChATGAL4/ + and UAS-InR^DN^/ + controls. **(P)** Mean total distance walked vs. age of TdcGAL4/UAS-InR^DN^ flies compared to TdcGAL4/ + and UAS-InR^DN^/ + controls. **(Q)** Mean duration of walking vs. age of TdcGAL4/UAS-InR^DN^ flies compared to TdcGAL4/ + and UAS-InR^DN^/ + controls. **(R)** Mean speed of walking vs. age of TdcGAL4/UAS-InR^DN^ flies compared to tdcGAL4/ + and UAS-InR^DN^/ + controls.

**FIGURE 4:**
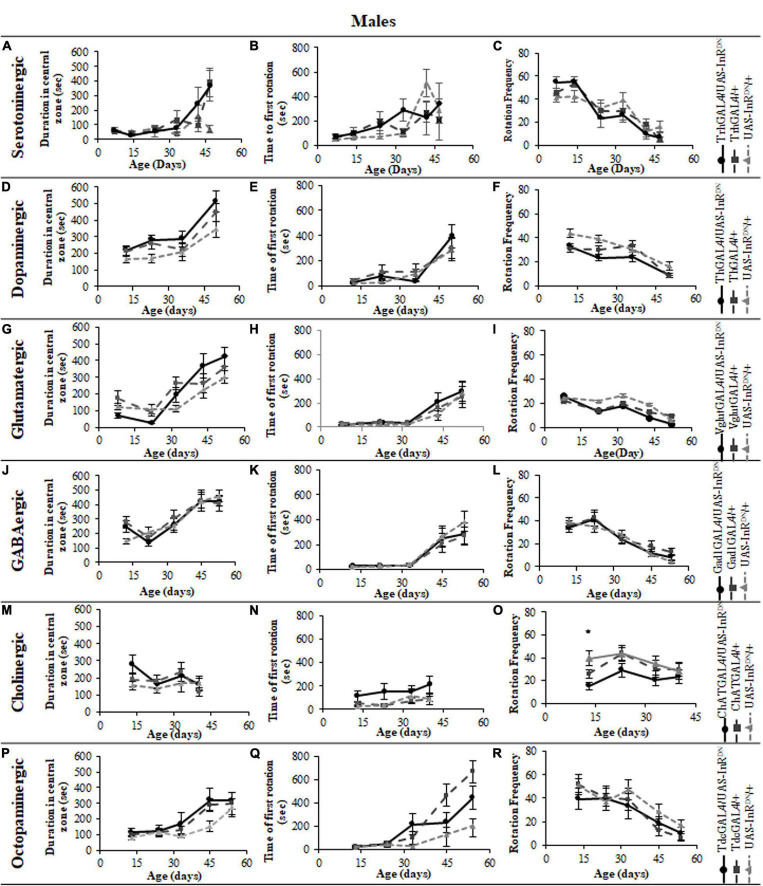
The effect of neuronal subtype specific IIS reduction on decision making during exploratory walking senescence in female flies. Exploratory walking senescence parameters for a cohort of female flies of the indicated genotypes run in parallel with the survival experiments shown in Figure 1. Data are shown as mean value for each parameter ± SEM, *N* = 16 for the indicated genotype. Data were analyzed by two-way ANOVA (age and genotype) and age was found to be a significant effect (*p* < 0.05) for all genotypes. **(A)** Mean duration flies spent in the central zone vs. age of TrhGAL4/UAS-InR^DN^ flies compared to TrhGAL4/ + and UAS-InR^DN^/ + controls. **(B)** Mean latency to first rotation vs. age of TrhGAL4/UAS-InR^DN^ flies compared to TrhGAL4/ + and UAS-InR^DN^/ + controls. **(C)** Mean rotation frequency vs. age of TrhGAL4/UAS-InR^DN^ flies compared to TrhGAL4/ + and UAS-InR^DN^/ + controls. **(D)** Mean duration flies spent in the central zone vs. age of ThGAL4/UAS-InR^DN^ flies compared to ThGAL4/ + and UAS-InR^DN^/ + controls. **(E)** Mean latency to first rotation vs. age of ThGAL4/UAS-InR^DN^ flies compared to ThGAL4/ + and UAS-InR^DN^/ + controls. **(F)** Mean rotation frequency vs. age of ThGAL4/UAS-InR^DN^ flies compared to ThGAL4/ + and UAS-InR^DN^/ + controls. **(G)** Mean duration flies spent in the central zone vs. age of VglutGAL4/UAS-InR^DN^ flies compared to VglutGAL4/ + and UAS-InR^DN^/ + controls. **(H)** Mean latency to first rotation vs. age of VglutGAL4/UAS-InR^DN^ flies compared to VglutGAL4/ + and UAS-InR^DN^/ + controls. **(I)** Mean rotation frequency vs. age of VglutGAL4/UAS-InR^DN^ flies compared to VglutGAL4/ + and UAS-InR^DN^/ + controls. **(J)** Mean duration flies spent in the central zone vs. age of Gad1GAL4/UAS-InR^DN^ flies compared to Gad1GAL4/ + and UAS-InR^DN^/ + controls. **(K)** Mean latency to first rotation vs. age of Gad1GAL4/UAS-InR^DN^ flies compared to Gad1GAL4/ + and UAS-InR^DN^/ + controls. **(L)** Mean rotation frequency vs. age of Gad1GAL4/UAS-InR^DN^ flies compared to Gad1GAL4/ + and UAS-InR^DN^/ + controls. **(M)** Mean duration flies spent in the central zone vs. age of ChATGAL4/UAS-InR^DN^ flies compared to ChATGAL4/ + and UAS-InR^DN^/ + controls. **(N)** Mean latency to first rotation vs. age of ChATGAL4/UAS-InR^DN^ flies compared to ChATGAL4/ + and UAS-InR^DN^/ + controls. **(O)** Mean rotation frequency vs. age of ChATGAL4/UAS-InR^DN^ flies compared to ChATGAL4/ + and UAS-InR^DN^/ + controls. **(P)** Mean duration flies spent in the central zone vs. age of TdcGAL4/UAS-InR^DN^ flies compared to TdcGAL4/ + and UAS-InR^DN^/ + controls. **(Q)** Mean latency to first rotation vs. age of TdcGAL4/UAS-InR^DN^ flies compared to TdcGAL4/ + and UAS-InR^DN^/ + controls. **(R)** Mean rotation frequency vs. age of TdcGAL4/UAS-InR^DN^ flies compared to TdcGAL4/ + and UAS-InR^DN^/ + controls.

**FIGURE 5:**
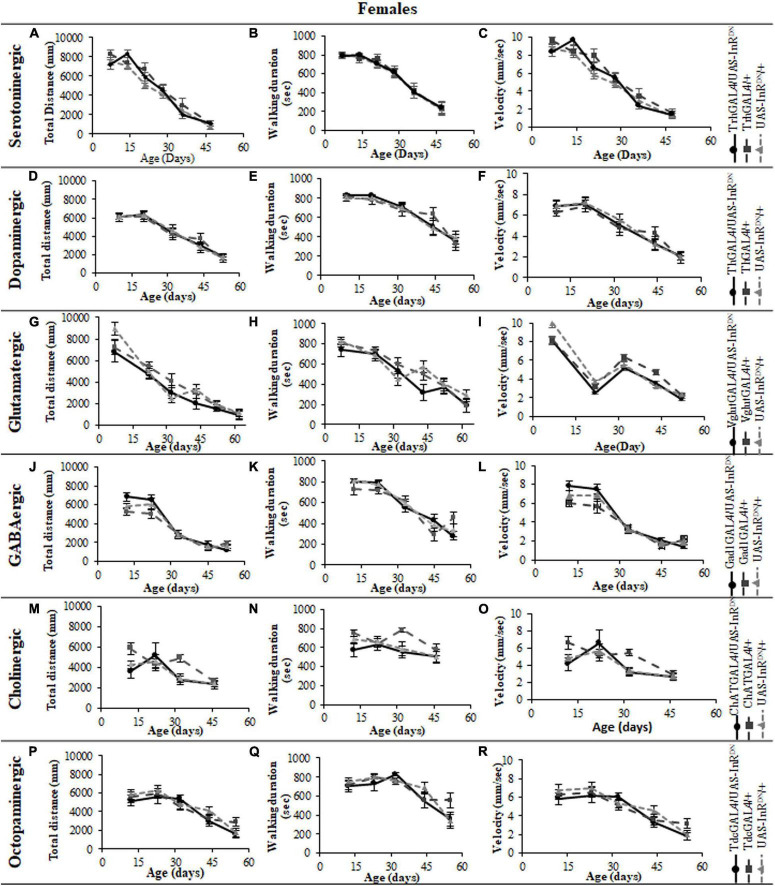
The effect of neuronal subtype specific IIS reduction on locomotion during exploratory walking senescence in male flies. Exploratory walking senescence parameters for a cohort of male flies of the indicated genotypes run in parallel with the survival experiments shown in Figure 1. Data are shown as mean value for each parameter ± SEM, *N* = 16 for the indicated genotype. Data were analyzed by two-way ANOVA (age and genotype) and age was found to be a significant effect (*p* < 0.05) for all genotypes. **(A)** Mean total distance walked vs. age of TrhGAL4/UAS-InR^DN^ flies compared to TrhGAL4/ + and UAS-InR^DN^/ + controls. **(B)** Mean duration of walking vs. age of TrhGAL4/UAS-InR^DN^ flies compared to TrhGAL4/ + and UAS-InR^DN^/ + controls. **(C)** Mean speed of walking vs. age of TrhGAL4/UAS-InR^DN^ flies compared to TrhGAL4/ + and UAS-InR^DN^/ + controls. **(D)** Mean total distance walked vs. age of ThGAL4/UAS-InR^DN^ flies compared to ThGAL4/ + and UAS-InR^DN^/ + controls. **(E)** Mean duration of walking vs. age of ThGAL4/UAS-InR^DN^ flies compared to ThGAL4/ + and UAS-InR^DN^/ + controls. **(F)** Mean speed of walking vs. age of ThGAL4/UAS-InR^DN^ flies compared to ThGAL4/ + and UAS-InR^DN^/ + controls. **(G)** Mean total distance walked vs. age of VglutGAL4/UAS-InR^DN^ flies compared to VglutGAL4/ + and UAS-InR^DN^/ + controls. **(H)** Mean duration of walking vs. age of VglutGAL4/UAS-InR^DN^ flies compared to VglutGAL4/ + and UAS-InR^DN^/ + controls. **(I)** Mean speed of walking vs. age of VglutGAL4/UAS-InR^DN^ flies compared to VglutGAL4/ + and UAS-InR^DN^/ + controls. **(J)** Mean total distance walked vs. age of Gad1GAL4/UAS-InR^DN^ flies compared to Gad1GAL4/ + and UAS-InR^DN^/ + controls. **(K)** Mean duration of walking vs. age of Gad1GAL4/UAS-InR^DN^ flies compared to Gad1GAL4/ + and UAS-InR^DN^/ + controls. **(L)** Mean speed of walking vs. age of Gad1GAL4/UAS-InR^DN^ flies compared to Gad1GAL4/ + and UAS-InR^DN^/ + controls. **(M)** Mean total distance walked vs. age of ChATGAL4/UAS-InR^DN^ flies compared to ChATGAL4/ + and UAS-InR^DN^/ + controls. At the age of 13 days ChATGAL4/UAS-InR^DN^ showed decreased walking duration compared to the controls (*P* = 0.0011 to ChATGAL4/ + and *P* = 0.0001 to UAS-InR^DN^/ +). **(N)** Mean duration of walking vs. age of ChATGAL4/UAS-InR^DN^ flies compared to ChATGAL4/ + and UAS-InR^DN^/ + controls. At the age of 13 days ChATGAL4/UAS-InR^DN^ showed decreased walking duration compared to the controls (*P* = 0.0011 to ChATGAL4/ + and *P* = 0.0001 to UAS-InR^DN^/ +). **(O)** Mean speed of walking vs. age of ChATGAL4/UAS-InR^DN^ flies compared to ChATGAL4/ + and UAS-InR^DN^/ + controls. At the age of 13 days ChATGAL4/UAS-InR^DN^ showed decreased speed of walking compared to the controls (*P* = 0.0045 to ChATGAL4/ + and *P* < 0.0001 to UAS-InR^DN^/ +). **(P)** Mean total distance walked vs. age of TdcGAL4/UAS-InR^DN^ flies compared to TdcGAL4/ + and UAS-InR^DN^/ + controls. **(Q)** Mean duration of walking vs. age of TdcGAL4/UAS-InR^DN^ flies compared to TdcGAL4/ + and UAS-InR^DN^/ + controls. **(R)** Mean speed of walking vs. age of TdcGAL4/UAS-InR^DN^ flies compared to tdcGAL4/ + and UAS-InR^DN^/ + controls.

**FIGURE 6:**
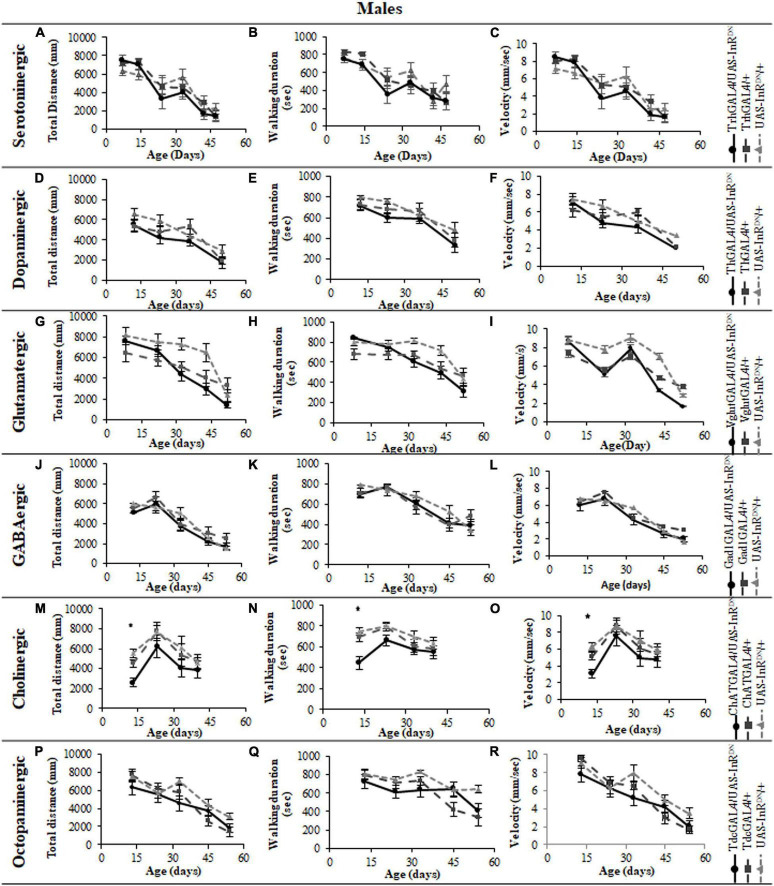
The effect of neuronal subtype specific IIS reduction on decision making during exploratory walking senescence in male flies. Exploratory walking senescence parameters for a cohort of male flies of the indicated genotypes run in parallel with the survival experiments shown in Figure 1. Data are shown as mean value for each parameter ± SEM, *N* = 16 for the indicated genotype. Data were analyzed by two-way ANOVA (age and genotype) and age was found to be a significant effect (*p* < 0.05) for all genotypes. **(A)** Mean duration flies spent in the central zone vs. age of TrhGAL4/UAS-InR^DN^ flies compared to TrhGAL4/ + and UAS-InR^DN^/ + controls. **(B)** Mean latency to first rotation vs. age of TrhGAL4/UAS-InR^DN^ flies compared to TrhGAL4/ + and UAS-InR^DN^/ + controls. **(C)** Mean rotation frequency vs. age of TrhGAL4/UAS-InR^DN^ flies compared to TrhGAL4/ + and UAS-InR^DN^/ + controls. **(D)** Mean duration flies spent in the central zone vs. age of ThGAL4/UAS-InR^DN^ flies compared to ThGAL4/ + and UAS-InR^DN^/ + controls. **(E)** Mean latency to first rotation vs. age of ThGAL4/UAS-InR^DN^ flies compared to ThGAL4/ + and UAS-InR^DN^/ + controls. **(F)** Mean rotation frequency vs. age of ThGAL4/UAS-InR^DN^ flies compared to ThGAL4/ + and UAS-InR^DN^/ + controls. **(G)** Mean duration flies spent in the central zone vs. age of VglutGAL4/UAS-InR^DN^ flies compared to VglutGAL4/ + and UAS-InR^DN^/ + controls. **(H)** Mean latency to first rotation vs. age of VglutGAL4/UAS-InR^DN^ flies compared to VglutGAL4/ + and UAS-InR^DN^/ + controls. **(I)** Mean rotation frequency vs. age of VglutGAL4/UAS-InR^DN^ flies compared to VglutGAL4/ + and UAS-InR^DN^/ + controls. **(J)** Mean duration flies spent in the central zone vs. age of Gad1GAL4/UAS-InR^DN^ flies compared to Gad1GAL4/ + and UAS-InR^DN^/ + controls. **(K)** Mean latency to first rotation vs. age of Gad1GAL4/UAS-InR^DN^ flies compared to Gad1GAL4/ + and UAS-InR^DN^/ + controls. **(L)** Mean rotation frequency vs. age of Gad1GAL4/UAS-InR^DN^ flies compared to Gad1GAL4/ + and UAS-InR^DN^/ + controls. **(M)** Mean duration flies spent in the central zone vs. age of ChATGAL4/UAS-InR^DN^ flies compared to ChATGAL4/ + and UAS-InR^DN^/ + controls. **(N)** Mean latency to first rotation vs. age of ChATGAL4/UAS-InR^DN^ flies compared to ChATGAL4/ + and UAS-InR^DN^/ + controls. **(O)** Mean rotation frequency vs. age of ChATGAL4/UAS-InR^DN^ flies compared to ChATGAL4/ + and UAS-InR^DN^/ + controls. At the age of 13 days ChATGAL4/UAS-InR^DN^ showed decreased rotation frequency compared to the controls (*P* = 0.0193 to ChATGAL4/ + and *P* < 0.0001 to UAS-InR^DN^/ +). **(P)** Mean duration flies spent in the central zone vs. age of TdcGAL4/UAS-InR^DN^ flies compared to TdcGAL4/ + and UAS-InR^DN^/ + controls. **(Q)** Mean latency to first rotation vs. age of TdcGAL4/UAS-InR^DN^ flies compared to TdcGAL4/ + and UAS-InR^DN^/ + controls. **(R)** Mean rotation frequency vs. age of TdcGAL4/UAS-InR^DN^ flies compared to TdcGAL4/ + and UAS-InR^DN^/ + controls.

In contrast to its beneficial effect on female lifespan, reduced IIS in serotonergic neurons had no effect on the normal age-related declines (senescence) of negative geotaxis locomotor behavior (Figure 2) and all parameters of exploratory walking locomotor behavior (Figures 3–6) in both males and females. Thus, despite being long-lived, females with reduced IIS in serotonergic neurons showed a similar locomotor behavior senescence to that of normally lived males and control genotypes.

### Reduced Insulin/IGF-Like Signaling Only in Cholinergic Neurons Has Detrimental Effects on Locomotor Behavior

Despite the variable effects on lifespan of reduced IIS in other neuronal subtypes, the normal senescence of negative geotaxis locomotor behavior was not affected by reduced IIS in dopaminergic, glutamatergic, octopaminergic, and GABAergic neurons (Figure 2). In contrast, IIS reduction in cholinergic neurons detrimentally affected both male and female negative geotaxis behavior (Figure 2). The negative geotaxis performance index of females with reduced IIS in cholinergic neurons (ChATGAL4/InR^DN^) was normal at 10 days old but declined sooner than controls, showing a significantly lower PI than controls at 20 days old (Figure 2I). Male ChATGAL4/InR^DN^ flies were more severely affected than females, showing a significantly lower PI than controls at all ages (Figure 2J).

All parameters of exploratory walking behavior and their senescence in male and female flies with reduced IIS in octopaminergic, dopaminergic, glutamatergic, and GABAergic neurons did not differ significantly from controls (Figures 3–6). However, similarly to the effect on negative geotaxis locomotor behavior, exploratory walking behavior parameters were detrimentally affected in flies with reduced IIS in cholinergic neurons (Figures 3–6). Male flies expressing the UAS-InR^DN^ transgene in cholinergic neurons were greatly affected at young age, showing shorter walking distance, speed and duration of walking compared to controls at 10 days old (Figures 6M–O). Male ChAT-GAL4/UAS-InR^DN^ performance of these parameters largely recovered and declined similarly to controls from age 20 days. Interestingly, not all decision making parameters were affected. The duration of time spent in the central zone of the chamber and the latency to making a first change of walking direction (“Time of first rotation”) were unaffected, but ChAT-GAL4/UAS-InR^DN^ males performed fewer changes in direction (rotations) at 10 days old compared to controls, and their rotation frequency then largely recovered and was not significantly different to controls at older ages (Figures 4M–O). Females with reduced IIS in cholinergic neurons showed normal declines in all walking parameters compared to controls (Figures 3M–O, 5M–O).

## Discussion

Together, the data presented here indicate that individual neuronal subtypes respond differently to reduced IIS in their modulation of lifespan and locomotor behavioral function, with little correlation between lifespan and behavioral effects. Reduced IIS restricted to serotonergic neurons is sufficient to extend female lifespan, with no effect on locomotor behavior, identifying a novel role for the insulin receptor in these neurons in the modulation of lifespan.

We have previously shown that lifespan and behavioral senescence are independently regulated by the *Drosophila* insulin receptor, as lifespan extending reductions in IIS do not ameliorate all forms of locomotor senescence such that lifespan extension can occur in the presence of normal, ameliorated or reduced behavioral function (Ismail et al., 2015). Thus, pan-neural reduction of IIS is not beneficial to the neural circuitry underlying negative geotaxis and exploratory walking behavior with age, despite extending female lifespan (Ismail et al., 2015). In this present study we attempted to determine how reduced IIS in specific neuronal subtypes contributed to these phenotypes, hypothesizing that the survival and behavioral outcomes of pan-neural IIS reduction could be due to the sum of neutral, negative and positive effects on the aging and/or function of different neuronal subtypes. Together, the data presented here indicate that individual neuronal subtypes respond differently to a reduction in IIS and play independent roles in the modulation of lifespan and locomotor behavior senescence. However, it is important to note that the effect of reducing IIS in specific neurons on lifespan or performance of a behavior is dependent on whether or not: (1) the neurons in question can respond to IIS (i.e., in that they normally express the insulin receptor); (2) the neurons are involved in the circuitry controlling lifespan or the behavior in question; and (3) IIS modulates the function or aging of the neurons. In addition, the study uses only one transgene to lower IIS and assumes that expression of this UAS-InR^DN^ transgene results in a functional reduction in IIS in specific neuronal subtypes, based on our previous demonstration that the transgene reduces IIS when expressed ubiquitously (Ikeya et al., 2009). The UAS-InR^DN^ transgene has also been used as a tool to reduce IIS in many studies (for example Slack et al., 2011; Chambers et al., 2015; Ismail et al., 2015; Eschment et al., 2020; Sudhakar et al., 2020; Atilano et al., 2021). We have kept these caveats in mind in the following discussion of the role of IIS in specific neuronal subtypes on lifespan and locomotor behavior senescence (Ikeya et al., 2009).

Although pan-neural IIS reduction in *Drosophila* results in extension of female lifespan with no effect on male lifespan (Ismail et al., 2015), the data presented here show that reduced IIS in all but one specific neuronal subtype did not promote female longevity, and in fact often lifespan was shortened. For instance, selective IIS reduction in GABAergic and octopaminergic neurons had no effect on lifespan, but IIS reduction in dopaminergic, cholinergic, and glutamatergic neurons shortened lifespan. Reducing IIS specifically in serotonergic neurons, however, was sufficient to extend female lifespan. This lifespan extension of females due to expression of the UAS-InR^DN^ transgene in serotonergic neurons with no effect on male lifespan is the same phenotype seen with pan-neural IIS reduction (Ismail et al., 2015). Given that the reduction of IIS in other neuronal subtypes did not extend female lifespan, the data presented here strongly suggest that the lifespan extension of females with pan-neural IIS reduction was due to the effect of UAS-InR^DN^ expression specifically in serotonergic neurons. This finding is interesting given the known role of serotonin in modulating lifespan in response to the sensory perception of diet and putative danger signals in the environment (Miller et al., 2020) (for review). The insulin producing cells (IPCs) in the fly brain that are involved in the response of lifespan to dietary restriction (DR) (Broughton et al., 2010) express the 5-HT 1A receptor (Luo et al., 2012). Serotonin via the 5-HT 2A receptor in the IPCs is involved in the modulation of lifespan in response to the perception of the protein value of the diet (Miller et al., 2020; Lyu et al., 2021). Neuronal expression of the serotonin 5-HT2A receptor is required for the lifespan effects of dietary choice in *Drosophila* and serotonergic activity within the CNS has been suggested to be a key regulator of aging and physiology in an environment where dietary choice is available (Lyu et al., 2021). Serotonin signaling in *C elegans* has been similarly implicated in modulating lifespan. For instance, it has been suggested that serotonin antagonistically modulates lifespan via different serotonin receptors (Murakami and Murakami, 2007) and more recently it has been shown that serotonin signaling is involved in the modulation of lifespan in response to environmental temperature via brain-gut communications (Zhang et al., 2018). Thus, although the mechanism of lifespan extension involved in our study is yet to be determined, our data identify a novel role for the *Drosophila* insulin receptor in serotonergic neurons in the modulation of lifespan. It will be interesting in future experiments to elucidate the potential role of diet in the modulation of lifespan by IIS in serotonin neurons. The role of IIS in serotonergic neurons in lifespan that we have identified here confirms that these neurons respond to altered IIS. Although it has been shown that serotonergic neurons in the ventral nerve chord (VNC) modulate walking speed under various conditions (Howard et al., 2019), long-lived flies with reduced IIS in serotonergic neurons showed no locomotor behavioral phenotypes in our study. It should be noted, however, that behavioral measurements were not made past the 47 day age point in the serotonergic experiment because walking speed, distance and rotation frequency of all genotypes had declined to very low levels by this age. It is thus possible that other parameters such as duration in the central zone could show differences between genotypes at older ages. Taken together, the data suggest that either IIS does not influence serotonergic neurons’ role in modulating walking speed or that the level of IIS reduction sufficient to modulate lifespan was not sufficient to alter the function of the serotonergic neurons themselves in modulating walking speed. Further studies are needed to determine if altered IIS in serotonergic neurons modulates serotonin levels and/or activity of the neurons.

Although we have identified a specific role for IIS in serotonergic neurons in the modulation of lifespan in *Drosophila*, the role of IIS in other neuronal subtypes is less clear. An extension of lifespan indicates a beneficial effect on organismal aging, but a shortened lifespan suggests either a detrimental effect on aging (promotion of aging) or a detrimental effect on a specific function that limits lifespan. In addition, each of the lifespan and behavioral experiments in the different neuronal subtypes in this study were performed at different times because the size of the experiments precluded running all in parallel. Thus, there is some variability in median lifespan of the UAS-InR^DN^/ + control genotype between experiments. Such lifespan variation is common in *Drosophila* and the median lifespan of the w*^Dah^* genetic background has been shown to vary from 32 to 75 days between experiments under defined control conditions (Ziehm et al., 2013). Similarly, inter-individual variability in behavior is common and well investigated (Molla-Albaladejo and Sanchez-Alcaniz, 2021), with trial to trial variability arising from noise in the nervous system (Faisal et al., 2008). Variability in exploratory walking parameters with age between experiments can be seen in our data when comparing the UAS-InR^DN^/ + genotype which was run in all experiments. As a result, the direct comparison of absolute median lifespans and behavioral performance values resulting from reduced IIS in different neuronal subtypes cannot be made and only comparisons between experimental and control genotypes within an individual experiment can be made in this study.

The detrimental effect of reduced IIS in cholinergic neurons on male lifespan, negative geotaxis and exploratory walking suggests that reduced IIS is not beneficial to the function of these neurons or to lifespan. Moreover, the detrimental effect on locomotor behavior at young age followed by recovery and a relatively normal age-related decline in function suggests that these detrimental behavioral outcomes are not due to an effect on neuronal aging and raise the possibility that the detrimental effects are due to a sex-specific effect of reduced IIS on cholinergic neuronal function or development. Our findings are interesting given that changes in cholinergic neurons are associated with aging (Schliebs and Arendt, 2011). Our data are also consistent with a recent finding that reduced function of the vesicular acetylcholine transporter (VAChT) results in reduced lifespan and defects in locomotor behavior (White et al., 2020). Importantly, we have identified the involvement of the *Drosophila* InR in these cholinergic neurons in the modulation of locomotor behavior, and possibly lifespan, and future studies are needed to determine if the InR mediates these effects via a modulation of the VAChT or other aspects of cholinergic neuron function. It should be noted, however, that the ChATGAL4/ + control genotype was short-lived in both sexes compared to the UAS-InR^DN^/ + control genotype likely indicating a negative effect of the GAL4 transgene insertion on the health of the flies that is limiting to lifespan. Despite this, ChATGAL4/UAS-InR^DN^ males were significantly shorter lived than both controls suggesting a further detrimental effect of reduced IIS in cholinergic neurons on male lifespan. However, given the lack of further detrimental effect of IIS reduction on female lifespan, an alternative interpretation is that the reduced survival of the ChATGAL4/UAS-InR^DN^ males is simply due to the ChATGAL4 transgene expression itself such that further studies using alternative GAL4 lines are necessary to confirm what effect, if any, reduced IIS in cholinergic neurons has on lifespan. Although the effect on lifespan is still unclear it is interesting that the ChATGAL4/ + driver did not induce a similar detrimental effect on locomotor behavior. In all behavioral measures, the ChATGAL4/ + control flies performed similarly to the UAS-InR^DN^/ + control flies despite being shorter lived indicating the disconnection between lifespan and behavioral senescence.

Reduced IIS in dopaminergic and glutamatergic neurons similarly resulted in a reduction in both male and female lifespan but had no significant effect on locomotor behaviors or their senescence. That reduced IIS in dopaminergic and glutamatergic neurons shortened lifespan indicates that these neuronal subtypes can respond to IIS modulation. Interestingly, although dopaminergic neurons are involved in the control of locomotor behavior including negative geotaxis behavior (Yellman et al., 1997; Lima and Miesenbock, 2005; Sun et al., 2018), reduced IIS in these neurons had no effect on negative geotaxis or exploratory walking behavior. This suggests that IIS is not involved in the modulation of the function of dopaminergic neurons in regulating locomotor behavior or that the level of IIS reduction induced in our model was not sufficient to alter the function of the neurons themselves in modulating locomotor behavior. However, the level of downregulation of IIS was sufficient to shorten lifespan, although the mechanism remains to be determined. In other studies, a lower dopamine metabolism plus higher antioxidant activity resulted in extended lifespan (Perez et al., 2021). A complete lack of dopamine was found to have no effect on lifespan (Riemensperger et al., 2011) which may have resulted from adaptive changes in the nervous system (Simon et al., 2009). It will be interesting to determine if dopamine levels are increased in short-lived flies with reduced IIS in dopamine neurons. For glutamatergic neurons, the mechanism mediating the lifespan shortening effect of reduced IIS seen here also remains to be determined. Glutamate neurons are sensitive to changes in IIS and glutamate antagonizes the neuroprotective role of IIS (Garcia-Galloway et al., 2003). However, a recent study raises the possibility that it may involve a modulation of glutamatergic neuron excitability. Wong et al. (2021) found that hyperexcitable glutamate neurons shortened *Drosophila* lifespan via aberrant Ca^2+^ signaling between the ER and lysosomes.

Selective IIS reduction in GABAergic and octopaminergic neurons had no effect on lifespan or locomotor senescence. Octopamine plays an important role in regulating aggression (Zhou et al., 2008; Andrews et al., 2014), it is necessary for adaptation to endurance exercise (Sujkowski et al., 2017) and it also regulates odor-based decision making (Classen and Scholz, 2018). Flies lacking octopamine are more resistant to starvation, have increased body fat deposit, reduced physical activity and reduced metabolic rate compared to the control flies (Li et al., 2016). These octopamine deficient flies have a shorter lifespan and increased rate of insulin release (Li et al., 2016). GABA is also involved in modulating multiple processes including aggression (Alekseyenko et al., 2019), sleep (Hamasaka et al., 2005), food consumption (Cheung and Scott, 2017), and appetitive long-term memory formation (Pavlowsky et al., 2018). GABA also mediates the behavior-impairing effect of ethanol (Dzitoyeva et al., 2003). Given the lack of effect of UAS-InR^DN^ expression in octopamine and GABA neurons in our study, it is likely that these neuronal subtypes are not sensitive to changes in IIS in relation to the modulation of lifespan and the control of the locomotor behaviors.

The phenotypes of flies with reduced IIS in cholinergic neurons seen here raises the possibility that the detrimental behavioral effects of pan-neural IIS reduction (Ismail et al., 2015) were due to the detrimental effect of reduced IIS specifically in cholinergic neurons. There are, however, differences in the lifespan and locomotor behavior phenotypes due to pan-neural (elavGAL4/UAS-InR^DN^) or cholinergic neuron-specific (ChATGAL4/UAS-InR^DN^) reduction in IIS. For instance, negative geotaxis senescence in flies with a pan-neural IIS reduction was normal (Ismail et al., 2015) but reduced IIS specifically in cholinergic neurons had a detrimental effect on this locomotor behavior. In addition, elavGAL4/UAS-InR^DN^ females were long-lived with no effect on male lifespan (Ismail et al., 2015) whereas ChATGAL4/UAS-InR^DN^ males were short-lived with no effect on female lifespan. In addition to the detrimental effect of the ChATGAL4 driver itself on lifespan discussed above, these differences in phenotypes may be due to differences in expression level of the GAL4 drivers in specific neuronal cell-types (elavGAL4 vs. ChATGAL4) resulting in a stronger or weaker reduction in IIS and/or compensatory effects in elavGAL4/UAS-InR^DN^ flies of reduced IIS in other neuronal subtypes (i.e., serotonergic neurons). These findings further highlight the complexity of IIS in the nervous system in aging. They also highlight that using lifespan as an indicator of the effects of a treatment on organismal aging, although useful, does not provide information about the animal’s health with age. Moreover, under controlled laboratory conditions animals can have normal or extended lifespans despite compromised health or function. In this study, we used two locomotor assays as measures of locomotor health and brain function with age in addition to measuring effects on aging using lifespan. Interestingly, for all neuronal subtypes other than cholinergic there was little correlation between lifespan and locomotor behavior effects of reduced neuronal IIS. Locomotor behaviors were largely unaffected by reduced IIS in dopaminergic, glutamatergic, GABAergic and serotonergic neurons despite the variable lifespan effects of reduced IIS in these neuronal subtypes. For instance, long-lived trhGAL4/UAS-InR^DN^ females (serotonergic) and short-lived ThGAL4/UAS-InR^DN^ (dopaminergic) and VglutGAL4/UAS-InR^DN^ (glutamatergic) flies, all showed a normal senescence of negative geotaxis and exploratory walking. IIS reduction in cholinergic neurons resulted in detrimental effects on locomotor behaviors and possibly shortened lifespan, the only instance of a similar effect on both lifespan and locomotor behavior. However, as mentioned above, although the short-lived ChATGAL4/UAS-InR^DN^ (cholinergic) experimental flies showed an exacerbated senescence of locomotor behavior compared to both controls (Figure 2), the ChATGAL4/ + controls showed a normal negative geotaxis senescence despite being shorter lived than the UAS-InR^DN^/ + control flies. Taken together, the data show that the disconnection between IIS modulated lifespan and behavioral health-span suggested in Ismail et al. (2015) exists at the level of individual neuronal subtypes.

## Conclusion

This study has shown that individual neuronal subtypes play specific and independent roles in the modulation of lifespan and locomotor behavioral senescence in response to altered IIS in *Drosophila*. In particular, we have identified a specific role for IIS in serotonergic neurons in extending lifespan, and IIS in cholinergic neurons in having negative effects on locomotor behavior. It is still unclear as to whether or not neuronal aging is altered in response to changes in IIS in each neuronal subtype. The data in other neuronal subtypes show neutral or detrimental effects on locomotor behavior in long or normally lived flies, which suggests that the aging of these neurons is not slowed or delayed by reduced IIS. However, there are detrimental behavioral effects of reduced IIS in these neuronal subtypes. Further studies are needed to elucidate the role of IIS in neuronal aging, and to identify the physiological, molecular, cellular, and possibly developmental, mechanisms mediating the detrimental effect of reduced IIS on behavioral function and the beneficial effect of reduced IIS in serotonergic neurons on longevity.

## Data Availability Statement

The original contributions presented in this study are included in the article/Supplementary Material, further inquiries can be directed to the corresponding author/s.

## Author Contributions

SB conceived and designed the experiments. SB, ND, TS, ID, CB, LO, GV, TW, and TT performed the experiments. SB and ND analyzed the data. SB, ND, and AS wrote the manuscript. All authors contributed to the article and approved the submitted version.

## Conflict of Interest

The authors declare that the research was conducted in the absence of any commercial or financial relationships that could be construed as a potential conflict of interest. Since 17th January 2022, the co-author GV has been employed by Frontiers Media SA. GV declared his/her affiliation with Frontiers, and the handling Editor states that the process nevertheless met the standards of a fair and objective review.

## Publisher’s Note

All claims expressed in this article are solely those of the authors and do not necessarily represent those of their affiliated organizations, or those of the publisher, the editors and the reviewers. Any product that may be evaluated in this article, or claim that may be made by its manufacturer, is not guaranteed or endorsed by the publisher.
