# Corrigendum: Reduced insulin signaling targeted to serotonergic neurons but not other neuronal subtypes extends lifespan in *Drosophila melanogaster*

**DOI:** 10.3389/fnagi.2023.1307495

**Published:** 2023-10-17

**Authors:** Nikolett Dravecz, Tommy Shaw, Isabella Davies, Casey Brown, Lewis Ormerod, Gin Vu, Tyler Walker, Taran Taank, Alan D. Shirras, Susan J. Broughton

**Affiliations:** Division of Biomedical and Life Sciences, Faculty of Health and Medicine, Lancaster University, Lancaster, United Kingdom

**Keywords:** ageing, behavioral senescence, insulin/IGF-like signaling, serotonergic neurons, *Drosophila*

In the published article, there was an error. An incorrect Drosophila stock reference description was included.

A correction has been made to the **Materials and Methods**, “*Fly Stocks and Maintenance*” section, paragraph 1. This sentence previously stated:

“UAS-dInRA1409K (chr. II) (denoted here as UAS-InR^DN^) was obtained from the Bloomington Drosophila Stock Centre (ref. FBal015635)”

The corrected sentence appears below:

“UAS-InR.K1409A (chr. II) (denoted here as UAS-InR^DN^) was obtained from the Bloomington Drosophila Stock Centre (8252)”

The authors apologize for this error and state that this does not change the scientific conclusions of the article in any way. The original article has been updated.

