# Supplementary material for: Reduced Insulin Signaling Targeted to Serotonergic Neurons but Not Other Neuronal Subtypes Extends Lifespan in Drosophila melanogaster
Source: Front Aging Neurosci. 2022 Jul 5;14:893444. doi: 10.3389/fnagi.2022.893444 (PMC9294736; doi:10.3389/fnagi.2022.893444)
Supplement: Supplementary file 1 [file Data_Sheet_1.PDF]

A

B

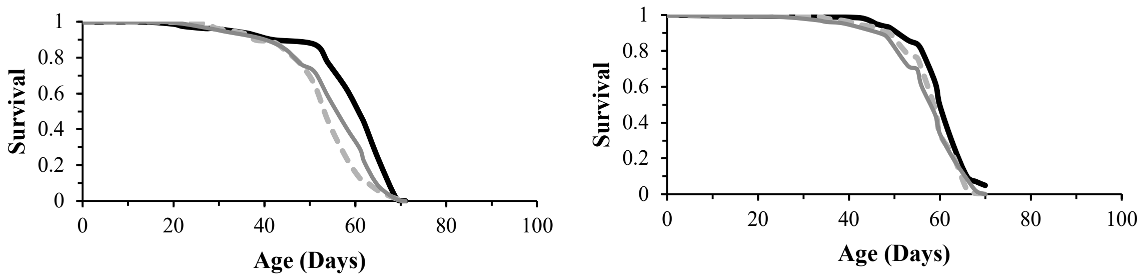


**Supplementary Figure. Survival of female flies with serotonergic neuron specific (TrhGAL4/UAS-InR$^{DN}$) reductions in IIS. (A) Cohort 1:** Survival of TrhGAL/UAS-InR$^{DN}$ once mated female flies compared to TrhGAL/+ and UAS-InR$^{DN}$/+ controls. Median lifespans and sample sizes were: TrhGAL/UAS-InR$^{DN}$ = 61 days, N=84; TrhGAL/+ = 54 days, N=91; and UAS-InR$^{DN}$ = 58 days, N=98. TrhGAL/UAS-InR$^{DN}$ females showed an increased survival compared to both controls by log rank tests (P<0.001). **(B) Cohort 2:** Survival of TrhGAL/UAS-InR$^{DN}$ once mated female flies compared to TrhGAL/+ and UAS-InR$^{DN}$/+ controls. Median lifespans and sample sizes were: TrhGAL/UAS-InR$^{DN}$ = 60 days, N=143; TrhGAL/+ = 59 days, N=160; and UAS-InR$^{DN}$ = 59 days, N=106. TrhGAL/UAS-InR$^{DN}$ females showed an increased survival compared to both controls by log rank tests (P=0.0017).
